# Supplementary material for: Abortive intussusceptive angiogenesis causes multi-cavernous vascular malformations
Source: eLife. 2021 May 20;10:e62155. doi: 10.7554/eLife.62155 (PMC8175082; doi:10.7554/eLife.62155)
Supplement: Supplementary file 1. [file elife-62155-supp1.docx]

Supplementary Table 1. Morpholino Sequences

| **Gene** | **Morpholino sequence (5’-3’)** | **concentration** | **reference** |
| --- | --- | --- | --- |
| *ccm2* | GAAGCTGAGTAATACCTTAACTTCC | 2ng/μl (fig.5), and 4ng/μl (Supp fig.4) | (Mably et al., 2006) |
| *tnnt2* | CATGTTTGCTCTGATCTGACACGCA | 5.3 ng/μl | (Sehnert et al., 2002) |
| *gata1* | CTGCAAGTGTAGTATTGAAGATGTC | 16ng/μl | (Galloway et al., 2005) |
| *tif1γ* | GCTCTCCGTACAATCTTGGCCTTTG | 2ng/μl | (Monteiro et al., 2011) |
| *klf2a* | GGACCTGTCCAGTTCATCCTTCCAC | 6ng/μl | (Nicoli et al., 2010) |
| *klf2b* | AAAGGCAAGGTAAAGCCATGTCCAC | 6ng/μl | (Nicoli et al., 2010) |
