# Supplementary figures and images for: Abortive intussusceptive angiogenesis causes multi-cavernous vascular malformations
Source: eLife. 2021 May 20;10:e62155. doi: 10.7554/eLife.62155 (PMC8175082; doi:10.7554/eLife.62155)

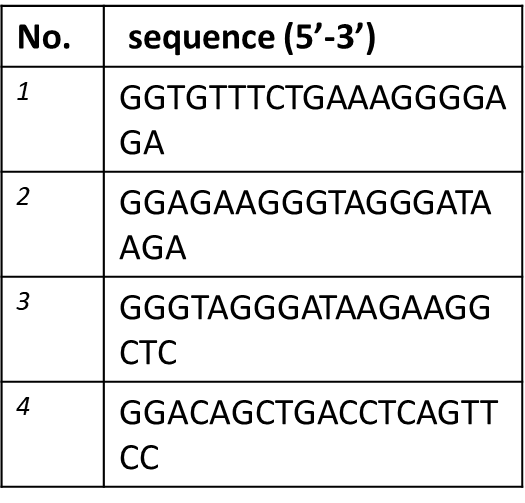


Supplementary Table 2. crRNA sequence for zebrafish *ccm2*

Supplement: Supplementary file 2. [file elife-62155-supp2.docx]
