# Supplementary material for: Abortive intussusceptive angiogenesis causes multi-cavernous vascular malformations
Source: eLife. 2021 May 20;10:e62155. doi: 10.7554/eLife.62155 (PMC8175082; doi:10.7554/eLife.62155)
Supplement: Supplementary file 3. [file elife-62155-supp3.docx]

Supplementary File 3: Primers for template DNA synthesis

|  | sequence (5’-3’) |
| --- | --- |
| morange forward | GAATACAAGCTACTTGTTCTTTTTGCAGGATCCCATCGATatggtgagcaagggcgagga |
| morange reverse | ATTCGCCGCTGCCCGCCGCGCTGCCCGCGCTGCCATCGATcttgtacagctcgtccatgc |
| ccm2 forward | CAGCGCGGGCAGCGCGGCGGGCAGCGGCGAATTTGAATTCatggaggaggatgtaaagaa |
| ccm2 reverse | GACTCACTATAGTTCTAGAGGCTCGAGAGGCCTTGAATTCtcaagatggcacgccgtctt |
| L197R forward | GTCTGCTGTCTGCTTGTGCGGGCTGTTGACAACAAGGCT |
| L197R reverse | AGCCTTGTTGTCAACAGCCCGCACAAGCAGACAGCAGAC |
| klf2a and Δklf2a forward | GGCGAATTTGAATTCATGGCTTTGAGTGGAACGATTT |
| klf2a reverse | AGAGGCCTTGAATTCCTACATATGACGTTTCATATGA |
| Δklf2a reverse | GAGAGGCCTTGAATTcCTAAGTCGCCGTGCGTTTCCTCGG |
| ccm2 probe forward | TAATACACTCACTATAGGGTCAAGATGGCACGCCGTCTT |
| ccm2 probe reverse | ATGGAGGAGGATGTAAAGAA |
| klf2a probe forward | TAATACGACTCACTATAGGGGTCCGTTCCTACATATGACGTTTC |
| klf2a probe reverse | GACTGGAAATGGCTTTGAGTGG |
